# Species-Specific Traits Rather Than Resource Partitioning Mediate Diversity Effects on Resource Use

**DOI:** 10.1371/journal.pone.0007423

**Published:** 2009-10-14

**Authors:** Jasmin A. Godbold, Rutger Rosenberg, Martin Solan

**Affiliations:** 1 Oceanlab, University of Aberdeen, Newburgh, Aberdeenshire, United Kingdom; 2 Department of Marine Ecology, Göteborg University, Fiskebäckskil, Sweden; Mt. Alison University, Canada

## Abstract

**Background:**

The link between biodiversity and ecosystem processes has firmly been established, but the mechanisms underpinning this relationship are poorly documented. Most studies have focused on terrestrial plant systems where resource use can be difficult to quantify as species rely on a limited number of common resources. Investigating resource use at the bulk level may not always be of sufficient resolution to detect subtle differences in resource use, as species-specific nutritional niches at the biochemical level may also moderate diversity effects on resource use.

**Methodology/Principal Findings:**

Here we use three co-occurring marine benthic echinoderms (*Brissopsis lyrifera*, *Mesothuria intestinalis*, *Parastichopus tremulus*) that feed on the same phytodetrital food source, to determine whether resource partitioning is the principal mechanism underpinning diversity effects on resource use. Specifically we investigate the use of phytodetrital pigments (chlorophylls and carotenoids) because many of these are essential for biological functions, including reproduction. Pigments were identified and quantified using reverse-phase high performance liquid Chromatography (HPLC) and data were analysed using a combination of extended linear regression with generalised least squares (GLS) estimation and standard multivariate techniques. Our analyses reveal no species-specific selectivity for particular algal pigments, confirming that these three species do not partition food resources at the biochemical level. Nevertheless, we demonstrate increased total resource use in diverse treatments as a result of selection effects and the dominance of one species (*B. lyrifera*).

**Conclusion:**

Overall, we found no evidence for resource partitioning at the biochemical level, as pigment composition was similar between individuals, which is likely due to plentiful food availability. Reduced intra-specific competition in the species mixture combined with greater adsorption efficiency and differences in feeding behaviour likely explain the dominant use of resources by *B. lyrifera*.

## Introduction

A wealth of theoretical and empirical studies has shown that changes in biodiversity can, irrespective of the ecosystem under study, affect the magnitude and direction of ecosystem processes [1], [2]. A clear understanding of the mechanisms that underpin this relationship, however, is still lacking and a source of continual debate [e.g. 3]–[5]. Several methodological approaches have been developed (e.g. overyielding [6]; additive partitioning [7]; tripartite partitioning [8]; diversity models [9]) to identify the mechanisms through which biodiversity modifies ecosystem function. Collectively, these distinguish between (1) the selection effect, which is the increased probability of including a functionally dominant species in diverse communities [6], [10], and (2) the complementarity effect, which includes resource partitioning and species facilitation [7]. A recent meta-analysis of mainly plant biodiversity experiments found that, in most studies, the relationships between biodiversity and ecosystem processes were driven by a combination of selection effects and complementarity effects, rather than by one mechanism alone [11].

Considering the importance of resource partitioning for species coexistence [12], [13], and the availability of statistical tools for determining its relative importance, it is surprising that there is still a lack of direct empirical evidence for resource partitioning as a mechanism through which biodiversity enhances ecosystem processes [14]. Whilst there is some indirect evidence for resource partitioning in aquatic systems, where the impact of consumer diversity exceeds that which can be explained by selection effects alone (e.g. [15], [16]), facilitative interactions may be more important in returning positive effects of species diversity (e.g. organic matter decomposition in fungal communities [17]). Large functional differences between species can lead to strong niche differentiation or facilitation, although these effects may not always be sufficient to result in strong overyielding or consistent increases in ecosystem function; diversity effects may, for example, depend on specific species combinations and environmental conditions [18].

It has been argued that the lack of evidence for resource partitioning in biodiversity experiments may be related to the difficulty of quantifying resource use, especially in plant systems where species depend on a limited number of common resources, such as light, water and nutrients [14]. Resource partitioning may, for example, be more easily detected in systems containing predators, where resource selectivity may be more apparent and therefore easier to quantify [19], [20]. However, resource partitioning has been detected between coexisting species at the macronutrient level; six generalist-feeding herbivores (grasshoppers) feeding on the same plant taxa consume protein and carbohydrate in different absolute amounts and ratios [13]. These species-specific nutritional niches moderate the effects of interspecific competition during periods of reduced resource quantity and quality and, therefore, may provide a mechanism by which overall resource use is increased in more diverse systems.

In marine benthic communities, seasonal and inter-annual variability in the quantity and quality of food supply is known to be a major structuring factor, especially in the deep sea [21], [22]. Yet, competition between deposit feeding benthic macrofauna was always thought to be low, which is likely due to individual species adopting different feeding strategies (e.g. particle size and patch selectivity or differences in mobility and feeding depth) that allow them to utilise different fractions of the same detrital food source [23]–[27]. Much of the evidence for resource partitioning, however, has mainly focussed on bulk level differences in resource use (e.g. sediment grain size or total organic carbon) that may not be of sufficient resolution to detect subtle differences in resource use. Recently, feeding selectivity has been demonstrated at the biochemical level using specific biomarkers, including fatty acids, sterols, and photosynthetic pigments (e.g. [28]–[31]). Photosynthetic pigments, such as chlorophyll and their degradation products, can be used as indicators of the quality of detrital material [31], whilst carotenoids form unique chemotaxonomic biomarkers of phytoplankton, macroalgae and seagrasses that can be used to identify sources of organic matter [32]–[34]. Carotenoids are particularly important for echinoderms because they are essential for many biological functions, including reproduction and defence mechanisms [35], [36] but, unlike prokaryotes, fungi, algae and higher plants, echinoderms cannot synthesise carotenoids *de novo* and therefore must obtain them from their diet. Here, we use photosynthetic biomarkers to investigate the effects of species diversity of three co-occurring echinoderm species (the sea urchin *Brissopsis lyrifera*, and the two sea cucumbers *Mesothuria intestinalis* and *Parastichopus tremulus*) that feed on the same phytodetrital resource. This is particularly important because deposit feeding organisms recycle and enrich localised areas of the seafloor through faecal pellet production which can influence faunal distribution and ecosystem functions, including nutrient cycling. Specifically, we investigate whether each species exhibits feeding selectivity for particular phytoplankton pigments (chlorophylls and/or carotenoids) and whether such partitioning of resources positively affects resource use when species are in mixture.

## Materials and Methods

Sediment and the deposit-feeding holothurians *Parastichopus tremulus* and *Mesothuria intestinalis,* and the echinoid *Brissopsis lyrifera*, were collected from two sites in the Gullmarfjord, Sweden (58°15.7′N 11°26.4′E and 58°22.1′N 11°34.3′E, depth 30–60 m), using a 1.5 m Agassiz trawl from the R.V. *Arne Tiselius*. Sediment from each trawl was sieved (500 µm) in a seawater bath to remove all macrofauna and allowed to settle (24 h) to retain the fine fraction (less than 63 µm). Sediment was homogenised to slurry (organic matter content, 6.98±0.52%) and distributed between aquaria (70×80×20 cm, n = 15; see Figure S1 in Supporting Information). To avoid effects of satiation and cross contamination of pigment signatures in faecal casts, individuals were starved for 24 h to evacuate the gut [37].

To simulate *in situ* conditions, aquaria were held in a constant temperature facility at 7.5±1°C in the dark. Each aquarium contained 20 L of sediment and had a continuous supply (1.33 L min^−1^) of deep (30 m) fjordic seawater. Replicate (n = 3) faunal communities were assembled in monoculture and in mixtures containing all three species (12 aquaria). Control aquaria without fauna (n = 3) were also assembled. Following [16], to ensure that any observed differences in resource use were due to species diversity effects, and not due to differences in the number of individuals feeding on the resource we adopted a substitutive design in which species density rather than biomass was kept constant between treatments (n = 3 individuals per aquarium). Controlling species density rather than biomass is preferable because the per capita biomass of the organisms used means that fine adjustment of biomass is not tractable. Instead, we controlled species density using similar sized organisms which also ensured that the densities of echinoderms were within the range typically observed in natural communities. The experiment ran for 3 days to ensure complete passage of sediment particles through the gut [24], [37], whilst also ensuring that resources remained available and were not depleted during the course of the experiment.

Sediment and faecal casts (*B. lyrifera*, n = 8; *M. intestinalis*, n = 27; *P. tremulus*, n = 28) were collected to establish the concentration and composition of photosynthetic pigments. In multi-species treatments, faecal casts from each individual species were not pooled to allow determination of species-specific pigment signatures when in mixture. The faecal casts were collected continuously throughout the experiment to avoid them being consumed by the echinoderms. All sediment samples were frozen at −80°C and freeze dried for pigment extraction.

The pigments were separated by ion pairing reverse phase HPLC, as described by [38] and modified by [39]. Pigments were extracted from 0.5 g freeze dried sediment in 3 ml of 90% HPLC grade acetone. The extracts were ultrasonicated for 2×30 seconds (Vibra Cell, Sonics & Materials Inc, Danbury, Conneticut, U.S.A.) and centrifuged at 3000 rpm for 10 minutes (Baird & Tatlock Auto Bench Centrifuge Mark IV). The supernatant (10 ml) from each sample was filtered through a 0.2 µm Nyalo membrane filter (Gelman) into amber vials and loaded into the chilled (4°C) HPLC autosampler tray. Sample aliquots (500 µl) were mixed with 1M ammonium acetate (500 µl) and 100 µl of the mixture was injected onto the HPLC column. The HPLC system (Thermo Finnigan Spectra System) was controlled by CHROMPAC (Thermoquest) software and included a Perkin Elmer C8 column. Carotenoids and chlorophylls were detected by absorbance at 440 nm and chlorophyll degradation products (phaeophytin *a* and phaeophorbide *a*) were detected by fluorescence at an excitation wavelength of 405 nm and an emission wavelength of 670 nm [40].

**Figure 1:**
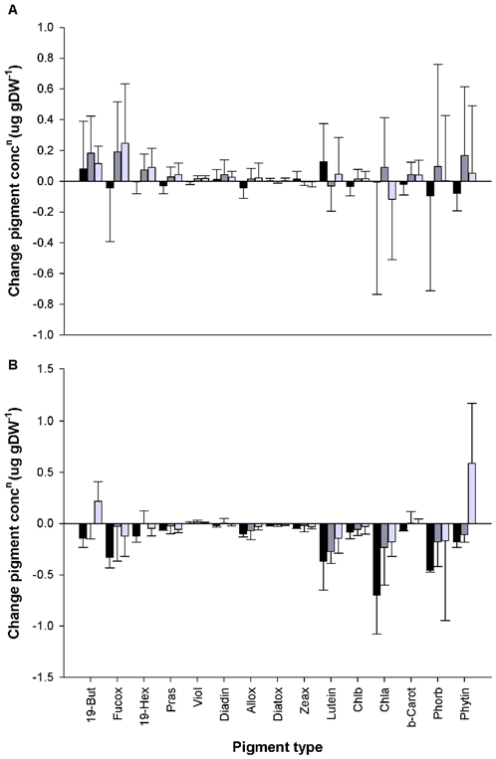
Mean change in total phytopigment concentration (µg gDW^−1^ ± SD) for echinoderm species in (a) monoculture and (b) mixture. Change is determined as differences in total pigment concentration between the initial background sediment and the faecal casts of *B. lyrifera* (black), *M. intestinalis* (dark grey) and *P. tremulus* (light grey) in monoculture and mixture. Abbreviations of the pigment types are: 19-But, 19 – Butanoyloxyfucoxanthin; Fucox, Fucoxanthin; 19-Hex, 19 – Hexanoyloxyfucoxanthin; Pras, Prasinoxanthin; Viol, Violaxanthin; Diadin, Diadinoxanthin; Allox, Alloxanthin; Diatox, Diatoxanthin; Zeax, Zeaxanthin; Lutein, Lutein; Chlb, Chlorophyll *b*; Chla, Chlorophyll *a*; b-Carot, β - Carotene; Phorb, Phaeophorbide *a*; Phytin, Phaeophytin *a*.

Pigments (n = 15, listed in the legend of Figure 1) were identified by comparing their individual retention times to those of commercially available pigment standards; Chlorophyll *a* and Chlorophyll *b* standards, Sigma Chemical Co. and a Pigmix standard, containing 20 pigments, Water Quality Institute (VKI), Hørsholm, Denmark. Identification was corroborated by comparing spectral data with these standards and by referring to the spectral information reported by [41].

Absolute pigment concentrations (µg g^−1^ sediment dry weight (DW)) of identified pigments were quantified as [32]:
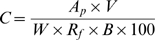



Where: *A_p_* is the peak area detected at 440 nm, *V* is the extract volume (ml), *W* is the dry weight of sediment (in grams), *R_f_* is the response factor and *B* is the buffer dilution factor (0.5). The response factors for each of the pigments were calculated by plotting concentrations of the standards against peak area.

We calculated the difference in pigment concentration between the faecal casts and background sediment for the total pigment concentration (change in total pigment concentration, ΔTPC µg gDW^−1^) and for each individual pigment (ΔPC µg gDW^−1^). A negative value for ΔTPC or ΔPC indicates that the faecal cast pigment concentration is lower than the background sediment. Statistical models were developed to investigate the effects of species identity (nominal explanatory variable, n = 5) on ΔTPC and ΔPC for each individual pigment. As the contribution of each species in mixture is not likely to be additive because species interact with one another (i.e. the presence of one species tends to alter the behaviour of another species, e.g. [42]), each species combination was treated as a unique ‘species’ identity [43].

Prior to the analyses, graphical exploratory techniques were used to check for homogeneity, normality and outliers of the data. Normality was determined by plotting the theoretical quantiles versus standardised residuals (Q-Q plots), while homogeneity of variance was evaluated by plotting residuals versus fitted values [44]. When model validation indicated normality, but heterogeneity of variances, relationships were defined using linear regression to which a generalised least squares estimation procedure [45] was applied, as detailed in [43]. Briefly, the use of GLS allows the variance structure imposed by the experimental design (large variances at low species richness levels and small variances at high species richness levels) to be modelled using variance functions (see [45]), avoiding the need for data transformation to homogenise the variance structure.

Differences in the phytopigment composition between species treatments were investigated using Gower's symmetrical dissimilarity coefficient for quantitative data [46] to calculate the dissimilarity matrix required for hierarchical cluster analysis (with group average linkage, [47]) and ANOSIM [48]. The dissimilarity matrix was based on ΔPC for each individual pigment (n = 15). Gower's coefficient is preferential to the more commonly used Bray-Curtis coefficient (e.g. [30], [49], [50]) for this type of biochemical data because it treats zeros and non-zeros in the same way and joint absences between treatments are incorporated into the dissimilarity matrix [47]. This is important as the presence/absence of a pigment may provide important information concerning biochemical differences between species. In addition, the importance of each pigment within the dissimilarity matrix is determined from its range of variation through all treatments [47], rather than giving greater weight to more common descriptors [44], [51].

In order to assess whether there were positive effects of species interactions on resource use, we compared the ΔTPC and ΔPC in species mixture to the best performing monoculture ( = overyielding [6]). As pigment concentrations in the faecal casts are expected to decrease as a result of echinoderm feeding, however, the appropriate reference response is the lowest value in monoculture. Thus, D_min_ was calculated as: 
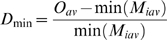



Where O*_av_* is the observed average ΔTPC or ΔPC (µg gDW^−1^) in the species mixture and min(M*_iav_*) the lowest average observed ΔTPC or ΔPC for species *i* monoculture. We conducted Monte Carlo simulations following the methods described by [52] to test whether D_min_ was significantly greater than zero for ΔTPC and ΔPC. The observed D_min_ was considered to be significantly greater than expected if there was no diversity effect, if the observed D_min_ was greater than the mean (±95% confidence interval) generated by the Monte Carlo simulations (one-tailed test with α = 0.05). We further determined the relative contribution of complementarity (CE) and selection effects (SE) to the observed net biodiversity effect (ΔY) using the additive partition equation of [7]. For comparative purposes, ΔY, CE and SE are multiplied by -1 to return positive values when positive effects are present.

All analyses were performed using the ‘vegan’ [53], ‘cluster’ [54] and ‘nlme’ [55] packages in the ‘R’ statistical and programming environment [56].

## Results

Fifteen phytoplankton pigments were identified from the HPLC chromatograms (listed in the legend of Figure 1). The pigment distribution in the faecal casts, irrespective of species identity, was similar to the background sediment (see Figure S2) and indicated that, at the time of the study, the sediments in the Gullmarfjord contain large quantities of fresh phytodetrital material (chlorophyll *a*: phaeophorbide  = 1.3) dominated by golden-brown flagellates (Haptophyta and Chrysophyta) and green algae (Chlorophyta) (see Table S1).

### Species identity effects on resource concentration

**Figure 2:**
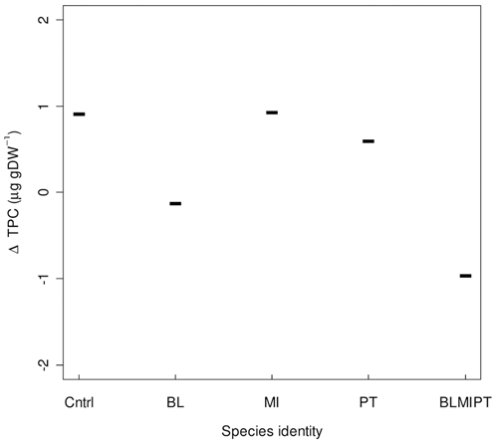
The effects of echinoderm species identity on the change in total phytopigment concentration (ΔTPC, µg gDW^−1^). Change is determined as differences in total pigment concentration between the faecal casts and the background sediment of *B. lyrifera* (BL), *M. intestinalis* (MI), *P. tremulus* (PT) and aquaria containing no macrofauna (CNTRL). Horizontal bars represent predicted values for each species identity. Individual data points are removed because the GLS analysis allows for differences in spread for species identity.

The effect of species identity on the ΔTPC (µg gDW^−1^) was analysed using a linear regression with GLS estimation and species identity as a variance covariate. The ΔTPC was affected by species identity (L-ratio  = 12.46, d.f. = 4, p<0.05) (Figure 2). When species were in mixture, the ΔTPC was more negative (i.e. lower pigment concentration in the faecal casts) in comparison to *M. intestinalis* (CV = −1.87±0.54 t = −3.466, p<0.001 [Bonferroni corrected, p<0.01]) and *P. tremulus* (CV = −1.55±0.50, t = −3.117, p<0.01 [Bonferroni corrected, p<0.05]) in monoculture, but not compared to *B. lyrifera* (CV = −0.85±0.80, t = −1.054, p = 0.296 [Bonferroni corrected, p = 1.0]). The observed result was driven by decreases in individual pigment concentrations (fucoxanthin, lutein, chlorophyll *a*, phaeophorbide), especially in the faecal casts of *B. lyrifera* (Figure 1).

### Species identity effects on resource composition

**Figure 3:**
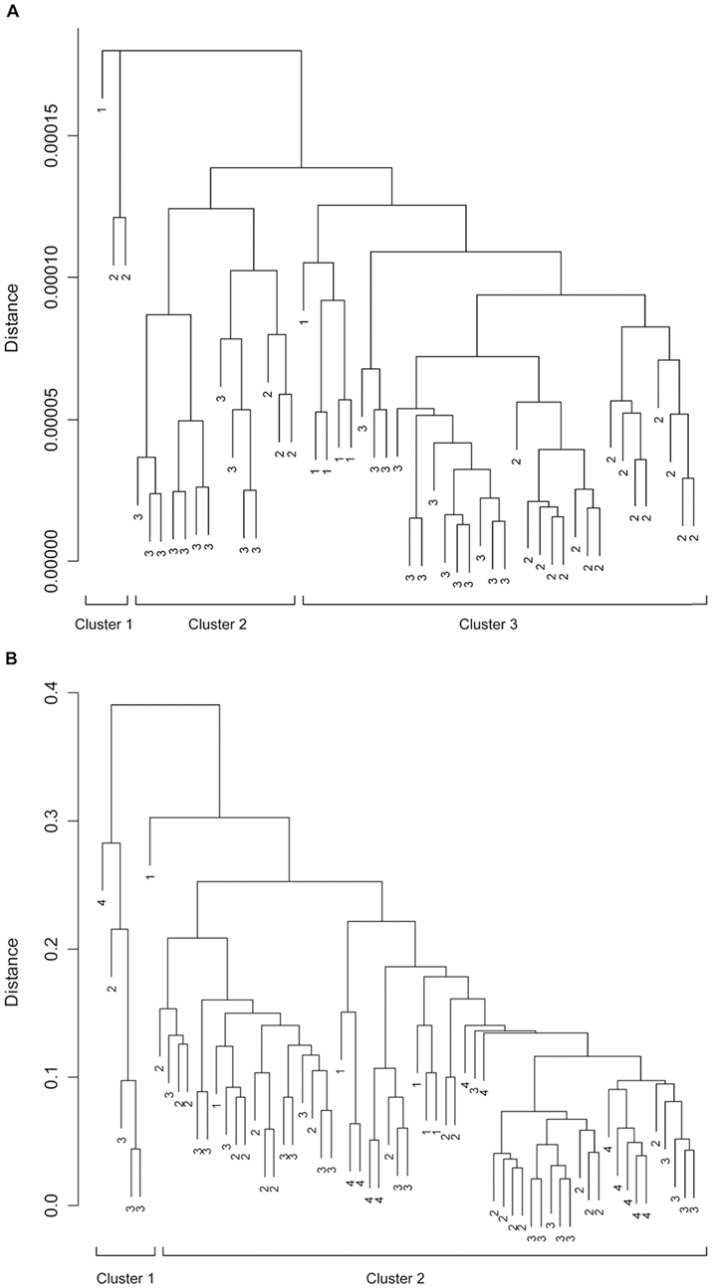
Hierarchical cluster analysis of change in phytopigment composition of species in (a) monoculture and (b) mixture. In (a) and (b) abbreviations are changes in pigment composition between the initial background sediment and the faecal casts of 1, *B. lyrifera*; 2, *M. intestinalis*; 3, *P. tremulus* in monoculture and in (b) 4, of the three species in mixture. Distance  =  dissimilarity in pigment composition between observations.

Cluster analysis revealed that differences in pigment composition between individuals in monoculture were subtle (3 clusters, distance  = 0.00013; Figure 3a) and because each cluster contained individuals from multiple species, pigment composition did not differ between species. There was no evidence for strong between-species variability for all quantified pigments (ANOSIM: global R = 0.481, p<0.001). There was also no evidence of differences in pigment composition between individuals in monoculture and individuals in the three species, as clusters contained individuals from all species in monoculture as well as the mixture (2 main clusters, distance  = 0.29; Figure 3b). ANOSIM analysis indicated that pigment profiles between individuals in monoculture and mixture were barely separable (ANOSIM: global R = 0.183, p<0.01).

### Overyielding and the net biodiversity effect

**Figure 4:**
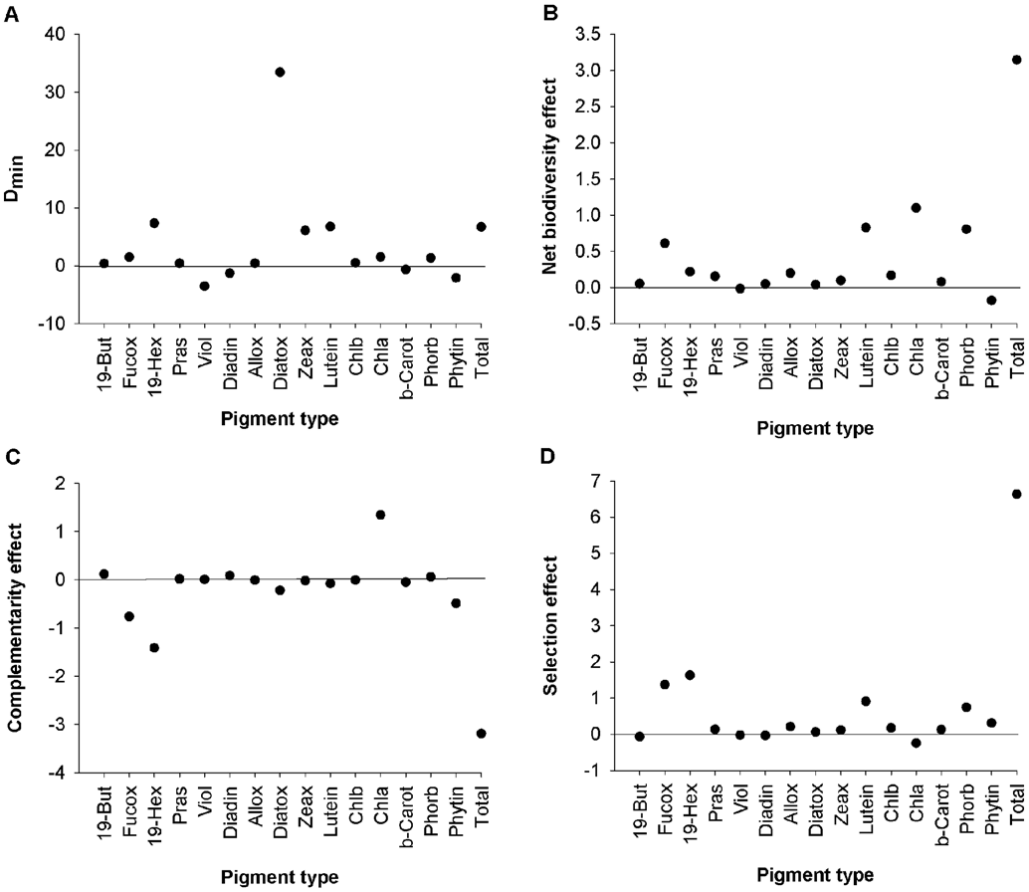
Summary of indices to identify the mechanisms through which echinoderm richness modifies phytopigment concentration. See Figure 1 for abbreviations of pigment type. Total represents the ΔTPC.

For ΔTPC there was evidence of overyielding and testing using Monte Carlo simulations revealed that the observed D_min_ (6.7) was significantly different from zero (i.e. significant overyielding). In 11/15 pigments the ΔPC in the species mixtures was more negative in comparison to the best performing monocultures, especially for 19-hexanoyloxyfucoxanthin (D_min_ = 7.3), diatoxanthin (D_min_ = 33.4), zeaxanthin (D_min_ = 5.0) and lutein (D_min_ = 6.8) (Figure 4a). Monte Carlo simulations confirmed that D_min_ for 9/15 pigments (11/15 when marginal results are included, p≤0.08) are significantly different from 0 (see Table S2).

The net biodiversity effect was positive for ΔTPC (ΔY = 3.145), with the observed response in the species mixture largely driven by the species with the highest effects on resource use in monoculture (SE = 6.634) and, to a lesser extent, by negative species interactions (CE = −3.489). The net biodiversity effects for ΔPC were generally positive, except for phaeophytin and, marginally, violaxanthin (Figure 4b). Biodiversity effects were higher (ΔY>0.5) for fucoxanthin, lutein, chlorophyll *a* and phaeophorbide relative to the remaining pigments. The relative contribution of SE and CE varied between individual pigments (Figure 4c, d). The positive ΔY, especially for fucoxanthin, lutein and phaeophorbide, was dominated by SE, indicating the dominance of a single species. In contrast, chlorophyll *a* was dominated by a positive CE which cancelled out the negative SE to give an overall positive ΔY. The remaining pigments showed weakly positive ΔY.

## Discussion

It is clear that species diversity positively affected resource use at the biochemical level, as there is evidence of overyielding for the majority of phytopigments. The observed net biodiversity effect was driven by the selection effect, suggesting increased resource use by a dominant species in mixture, a result consistent with many previous studies (for review, see [2]). However, an overall negative complementarity effect also contributed to the net effect of diversity, indicating the presence of negative (interference and/or exploitative) competition [57], [58] in the species mixture. Our data shows that this effect only occurred for a subset of pigments (fucoxanthin, 19-hexanoyloxyfucoxanthin and phaeophorbide; Figure 1) where the relative change in resource use by *M. intestinalis* and *P. tremulus* exceeded that of *B. lyrifera*. Thus the observed net effect of diversity resulted from species-specific selection effects associated with the competitive release of *B. lyrifera* and its subsequent dominance in the species mixture. This is best explained by the reduction in the negative effects of intra-specific competition associated with the lower densities of individual species when in species mixture [57], [59].

The change in pigment concentration between the faecal casts and sediment for *B. lyrifera* in monoculture was higher than that observed for monocultures of both *M. intestinalis* and *P. tremulus*. These patterns are likely due to inter-specific differences in feeding rates which ultimately affect gut residence time, and subsequently digestion and assimilation rates [60], [61]. As the gut residence time for *B. lyrifera* (19 to 75 hrs depending on location and conditions, [62]) is generally longer than that of *M. intestinalis* (∼23 hrs) or *P. tremulus* (∼12 hrs) [37], the process of digestion and assimilation may be strikingly different between species because the time available to breakdown and utilise organic compounds is extended [60]. Indeed, several studies have found that a slower feeding rate increases the gut residence time for food, which subsequently leads to greater absorption efficiency (e.g. [61]). Thus, a slower feeding rate and longer gut residence time is likely to enhance the absorption efficiency of *B. lyrifera* above that of *M. intestinalis* and *P. tremulus*, resulting in a more comprehensive use of the available labile organic material in *B. lyrifera*, but incomplete digestion and enhanced pigment concentrations in the faecal pellets of *M. intestinalis* and *P. tremulus*.

Interactions between species, resulting from competition for food and space or following disturbance and modification of the substratum (e.g. [63]), are important in regulating the structure and functioning of benthic communities. Species-specific strategies in terms of timing, spatial distribution or type of resource demand, will increase resource exploitation and result in a positive relationship between biodiversity and ecosystem function [12]. Similar to a recent study [64] in shallow shelf waters (<600 m depth), we also found a high degree of niche overlap in terms of resource use (all three echinoderm species utilise the same phytopigments) in our coastal system. It appears that feeding selectivity for labile organic material and biochemicals is more pronounced at greater water depths as a result of lower food inputs [49]. Thus, the lack of evidence for selective feeding for specific phytoplankton pigments in the present study may be explained by the more plentiful food available in coastal areas. The ready supply of organic material to the benthos may also decrease inter-specific competition for the food resource and hence reduce the potential for fine-scale niche separation [64]. Feeding selectivity in shallow water species has only been shown at the bulk level (fresh vs. old detritus) [26], [37] and not at the pigment level. *P. tremulus* and *M. intestinalis* have similar tentacular feeding structures and exhibit similar particle size selectivity, but *P. tremulus* feeds at rates 3 times faster than *M. intestinalis*. We contend, therefore, that competition between the species used in this study is reduced, at least in part, because of inter-specific differences in feeding and digestion rates [37], although we cannot discount the importance of the occupation of different sediment depth strata as a further mechanism of reducing inter-specific competition for space and resources [23]. Strong negative effects of species interactions on feeding and, subsequently, on growth and gonad production is common in benthic communities (e.g. [23], [24]). For example, the feeding and growth of the brittle star *Amphiura chiajei* can be depressed as a result of the physical disturbance caused by the burrowing activities of *B. lyrifera*, reducing its competitive ability to capture food [24].

It is important to consider the implications that changes in faecal cast phytopigment concentration and composition may have for other benthic fauna. The present findings, although weak, support previous views that holothurians may enrich localised areas of the seafloor by re-packing sediment into faecal material [65], [66]. In areas of localised and patchy inputs of organic matter, this may be especially important because changes in the sediment chemistry through faecal casts can have strong secondary effects on other benthic organisms (e.g. [63], [67]). Mobile fauna will rapidly move between organically enriched patches, process and re-distribute resources, thereby increasing the spatial heterogeneity of the system [63]. In addition, egestion of fresh faeces which are richer in organic content and generally have a smaller particle size than the surrounding sediment enhances bacterial biomass [68], and makes the faecal sediment nutritionally more attractive to other benthic deposit-feeders. In fact, faecal casts are the dominant food items in many holothurians (e.g. *P. tremulus* [69] and *Scotoplanes murrayi* [70]). The fact that phaeophorbide was among the dominant pigments in the faecal casts, also suggests that faecal material made up a large part of the ingested sediment. Feeding selectivity for faecal casts, organically enriched particles, or certain particle sizes has been found for many shallow–water echinoderms (e.g. [71]), but this ability is thought to vary between species and habitats. For example [37] detected feeding selectivity for organically enriched patches in *M. intestinalis* and *P. tremulus*, whilst [72] found no evidence of selection by particle size or for organically enriched particles in shallow water holothurians.

The presence of high concentrations of chlorophyll *a* in the gut sediments of the three species indicate that freshly deposited phytodetritus comprises a large part of the ingested material. Crucially, this fresh phytodetritus also contains large amounts of biochemical compounds, such as carotenoids, that can only be obtained from the diet, as they are not synthesised *de novo* [33] by echinoderms. Carotenoid pigments have fundamental biological functions as they have been found to increase, amongst others, the egg quality, larval quality and biological defence mechanisms in echinoderms [35], [36]. Overall 19-butanoxyfucoxanthin, fucoxanthin, lutein and 19- hexanoyloxyfucoxanthin were the dominant carotenoid pigments in the guts and strongly reduced in concentration relative to the background sediments. In the sea urchin *Lytechinus variegatus* the xanthophylls lutein and zeaxanthin were found to be more important for reproduction in terms of the number of juveniles produced and their survival rates than had previously been thought [73]. In addition, [35] reported that fucoxanthin, β-carotene and β-echinenone (not identified in the present study) enhanced biological defence reactions and also increased egg production in the sea urchin, *Pseudocentrotus depressus*. Therefore carotenoids are of vital importance for the fitness and reproductive success in echinoderms. Thus, species that can select and respond most quickly to high quality food input are likely to have a selective advantage [30].

### Conclusions

The present study was a direct experimental investigation into the mechanism(s) that underpin the biodiversity - ecosystem function relationship. There was a high degree of dietary niche overlap in terms of phytopigment use with no evidence of resource partitioning of the phytodetrital material at the biochemical level, most likely due to the plentiful availability of food in coastal areas. Consistent with the conclusion of several individual studies (see [2]) our results suggest that the observed net biodiversity effect is dominated by species-specific selection effects associated with the competitive release of a single species (*B. lyrifera*) when in mixture. In addition, physiological differences in adsorption efficiency and behavioural differences in feeding strategy can provide the mechanistic basis for species dominance and may be more important for resource use than resource partitioning in diverse communities.

## Supporting Information

Figure S1 Aquaria (randomly arranged) containing communities of *Parastichopus tremulus*, *Mesothuria intestinalis* and *Brissopsis lyrifera* in monoculture and in mixtures of three species in the temperature controlled room.
(0.69 MB TIF)

Figure S2 Mean pigment concentration (µg gDW -1±SD) of the background sediment (a), the faecal casts of *B. lyrifera* (black), *M. intestinalis* (dark grey) and *P. tremulus* (light grey) in monoculture. Abbreviations of the pigment types are: 19-But, 19-Butanoyloxyfucoxanthin; Fucox, Fucoxanthin; 19-Hex, 19-Hexanoyloxyfucoxanthin; Pras, Prasinoxanthin; Viol, Violaxanthin; Diadin, Diadinoxanthin; Allox, Alloxanthin; Diatox, Diatoxanthin; Zeax, Zeaxanthin; Lutein, Lutein; Chlb, Chlorophyll b; Chla, Chlorophyll a; b-Carot, β - Carotene; Phorb, Phaeophorbide a; Phytin, Phaeophytin a.
(0.17 MB TIF)

Table S1 Summary of the characteristic pigment biomarkers used for identification of the main phytoplankton phyla. Within the Phyla Chlorophyta and Haptophyta additional biomarkers allow identification of phytoplankton groups to Family level. Also included are the pigment sources of Chlorophyll breakdown products (compiled from Barlow et al. 1993a, Barlow et al. 1993b, Jeffrey 1997, Jeffrey et al. 1999, SchlÃ¼ter et al. 2000, Zapata et al. 2004).
(0.05 MB DOC)

Table S2 Summary of observed D_min_ indices of ΔTPC (Total) and ΔPC for each individual pigment and Monte Carlo simulations (mean ±95% confidence interval). If p<0.05 then the observed D_min_ was considered significantly greater than expected if there was no diversity effect. Abbreviations of the pigment types are: 19-But, 19-Butanoyloxyfucoxanthin; Fucox, Fucoxanthin; 19-Hex, 19-Hexanoyloxyfucoxanthin; Pras, Prasinoxanthin; Viol, Violaxanthin; Diadin, Diadinoxanthin; Allox, Alloxanthin; Diatox, Diatoxanthin; Zeax, Zeaxanthin; Lutein, Lutein; Chlb, Chlorophyll b; Chla, Chlorophyll a; b-Carot, β - Carotene; Phorb, Phaeophorbide a; Phytin, Phaeophytin a.
(0.04 MB DOC)
