# Supplementary figures and images for: Species-Specific Traits Rather Than Resource Partitioning Mediate Diversity Effects on Resource Use
Source: PLoS One. 2009 Oct 14;4(10):e7423. doi: 10.1371/journal.pone.0007423 (PMC2759289; doi:10.1371/journal.pone.0007423)

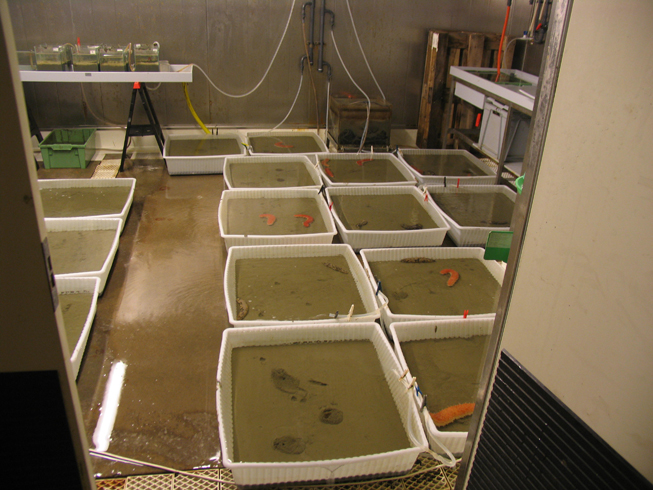

Supplement: Figure S1 — Aquaria (randomly arranged) containing communities of Parastichopus tremulus, Mesothuria intestinalis and Brissopsis lyrifera in monoculture and in mixtures of three species in the temperature controlled room. (0.69 MB TIF) [file pone.0007423.s001.tif]
