# Supplementary material for: Species-Specific Traits Rather Than Resource Partitioning Mediate Diversity Effects on Resource Use
Source: PLoS One. 2009 Oct 14;4(10):e7423. doi: 10.1371/journal.pone.0007423 (PMC2759289; doi:10.1371/journal.pone.0007423)
Supplement: Table S1 — Summary of the characteristic pigment biomarkers used for identification of the main phytoplankton phyla. Within the Phyla Chlorophyta and Haptophyta additional biomarkers allow identification of phytoplankton groups to Family level. Also included are the pigment sources of Chlorophyll breakdown products (compiled from Barlow et al. 1993a, Barlow et al. 1993b, Jeffrey 1997, Jeffrey et al. 1999, SchlÃ¼ter et al. 2000, Zapata et al. 2004). (0.05 MB DOC) [file pone.0007423.s003.doc]

| **Phytoplankton phyla or pigment source** | **Phytoplankton class** | **Characteristic pigment biomarkers** |
| --- | --- | --- |
| **Phylum Bacillariophyta** (diatoms) |  | Fucoxanthin, diadinoxanthin,  β-carotene, diatoxanthin |
| **Phylum Chlorophyta[[1]](#footnote-2)** (green algae) |  | Chlorophyll *b*, lutein, violaxanthin,  β-carotene, |
|  | Chlorophyceae | zeaxanthin |
|  | Prasinophyceae | prasinoxanthin |
|  |  | zeaxanthin |
| **Phylum Cryptophyta** (nanoplanktonic flagellates) |  | Alloxanthin |
| **Phylum Cyanophyta** (cyanobacteria) |  | Zeaxanthin, β-carotene |
| **Phylum Dinophyta** (dinoflagellates) |  | Diadinoxanthin, diatoxanthin,  β-carotene |
| **Phylum Euglenophyta** (fusiform flagellates) |  | Violaxanthin, zeaxanthin, β-carotene, diadinoxanthin, chlorophyll *b* |
| **Phylum Haptophyta[[2]](#footnote-3)** (golden-brown flagellates) |  | Fucoxanthin, diadinoxanthin,  β-carotene |
|  | Prymnesicacea | 19’- hexanoyloxyfucoxanthin |
|  |  | 19’- butanoyloxyfucoxanthin |
|  | Phaeocystaceae | 19’- hexanoyloxyfucoxanthin |
|  |  | 19’- butanoyloxyfucoxanthin |
|  |  | diatoxanthin |
| **Phylum Chrysophyta** (golden-brown flagellates) |  | 19’- butanoyloxyfucoxanthin,  β-carotene, fucoxanthin |
| **Zooplankton faecal pellets** (grazing) |  | Phaeophorbide *a*, phaeophytin *a* |
| **General breakdown product of Chlorophyll *a*** |  | Phaeophytin *a* |

**Table S1: Summary of the characteristic pigment biomarkers used for identification of the main phytoplankton phyla[[3]](#footnote-4).**

REFERENCES

Barlow, R.G., Mantoura, R.F.C., Gough, M.A. and Fileman, T.W. 1993a. Pigment signatures of the phytoplankton composition in the northeastern Atlantic during the 1990 spring bloom. *Deep-Sea Res. II* 40: 459-477.

Barlow, R.G., Mantoura, R.F.C., Gough, M.A. and Fileman, T.W. 1993b. Phaeopigment distribution during the 1990 spring bloom in the northeastern Atlantic. *Deep-Sea Res. II* 40: 2229-2242.

Jeffrey, S.W. 1997. Application of pigment methods to oceanography. In: Jeffrey, S.W., Mantoura, R.F.C. and Wright, S.W. (eds) Phytoplankton pigments in oceanography: guidelines to modern methods. UNESCO monographs on oceanographic methodology, 10. UNESCO, Paris. pp. 127-166.

Jeffrey, S.W., Wright, S.W. and Zapata, M. 1999. Recent advances in HPLC pigment analysis of phytoplankton. *Mar. Freshwater Res*. 50: 879-96.

Schlüter, L., Møhlenberg, F., Havskum, H. and Larsen, S. 2000. The use of phytoplankton pigments for identifying and quantifying phytoplankton groups in coastal areas: testing the influence of light and nutrients on pigment/chlorophyll a ratios. *Mar. Ecol. Prog. Ser*. 192: 49-63.

Zapata, M., Jeffrey, S.W., Wright, S.W., Rodríguez, F., Garrido, J.L. and Clementson, L. 2004. Photosynthetic pigments in 37 species (65 strains) of Haptophyta: implications for oceanography and chemotaxonomy. *Mar. Ecol. Prog. Ser*. 270: 83-102.

1. Additional biomarkers allow identification of phytoplankton groups to Family level [↑](#footnote-ref-2)
2. Additional biomarkers allow identification of phytoplankton groups to Family level [↑](#footnote-ref-3)
3. compiled from Barlow *et al.* 1993a, Barlow *et al.* 1993b, Jeffrey 1997, Jeffrey *et al.* 1999, Schlüter *et al.* 2000, Zapata *et al.* 2004 [↑](#footnote-ref-4)
