# Supplementary material for: Species-Specific Traits Rather Than Resource Partitioning Mediate Diversity Effects on Resource Use
Source: PLoS One. 2009 Oct 14;4(10):e7423. doi: 10.1371/journal.pone.0007423 (PMC2759289; doi:10.1371/journal.pone.0007423)
Supplement: Table S2 — Summary of observed Dmin indices of ΔTPC (Total) and ΔPC for each individual pigment and Monte Carlo simulations (mean ±95% confidence interval). If p<0.05 then the observed Dmin was considered significantly greater than expected if there was no diversity effect. Abbreviations of the pigment types are: 19-But, 19-Butanoyloxyfucoxanthin; Fucox, Fucoxanthin; 19-Hex, 19-Hexanoyloxyfucoxanthin; Pras, Prasinoxanthin; Viol, Violaxanthin; Diadin, Diadinoxanthin; Allox, Alloxanthin; Diatox, Diatoxanthin; Zeax, Zeaxanthin; Lutein, Lutein; Chlb, Chlorophyll b; Chla, Chlorophyll a; b-Carot, β - Carotene; Phorb, Phaeophorbide a; Phytin, Phaeophytin a. (0.04 MB DOC) [file pone.0007423.s004.doc]

| **Pigment type[[1]](#footnote-2)** | **Observed D_min_** | **mean (± 95% CI)** | **p-value[[2]](#footnote-3)** |
| --- | --- | --- | --- |
| **Total** | 6.7 | -1.4 (0.23) | <0.05 |
| **19-But** | 0.4 | 0.3 (5.9) | 0.480 |
| **Fucox** | 1.5 | -2.1 (0.6) | <0.01 |
| **19-Hex** | 7.3 | -2.3 (0.8) | <0.01 |
| **Pras** | 0.4 | -1.3 (0.5) | <0.01 |
| **Viol** | -3.5 | -3.6 (12.3) | 0.497 |
| **Diadin** | -1.3 | -0.7 (13.4) | 0.569 |
| **Allox** | 0.4 | -0.9 (0.1) | <0.05 |
| **Diatox** | 33.4 | -0.6 (4.3) | <0.01 |
| **Zeax** | 5.0 | -0.4 (2.0) | <0.01 |
| **Lutein** | 6.8 | -0.6 (1.0) | <0.0001 |
| **Chlb** | 0.5 | -0.9 (0.2) | <0.01 |
| **Chla** | 1.5 | -0.6 (1.3) | <0.05 |
| **b-Carot** | -0.6 | -1.7 (0.5) | 0.064 |
| **Phorb** | 1.3 | -0.9 (1.8) | 0.083 |
| **Phytin** | -2.1 | -1.7 (0.1) | 0.641 |

**Table S2: Summary of observed D_min_ indices of ∆TPC (Total) and ∆PC for each individual pigment and Monte Carlo simulations (mean ± 95% confidence interval).**

1. Abbreviation are: 19-But, 19 – Butanoyloxyfucoxanthin; Fucox, Fucoxanthin; 19-Hex, 19 – Hexanoyloxyfucoxanthin; Pras, Prasinoxanthin; Viol, Violaxanthin; Diadin, Diadinoxanthin; Allox, Alloxanthin; Diatox, Diatoxanthin; Zeax, Zeaxanthin; Lutein, Lutein; Chlb, Chlorophyll *b*; Chla, Chlorophyll *a*; b-Carot, β - Carotene; Phorb, Phaeophorbide *a*; Phytin, Phaeophytin *a*. [↑](#footnote-ref-2)
2. If p < 0.05 then the observed D_min_ was considered significantly greater than expected if there was no diversity effect. [↑](#footnote-ref-3)
